# Development and implementation of virtual instrumentation based on LabView applied to compression ignition engines operated with diesel-biodiesel blends

**DOI:** 10.1016/j.mex.2019.09.024

**Published:** 2019-10-04

**Authors:** Armando Pérez, Gisela Montero, Rogelio Ramos Irigoyen, Conrado Garcia, Marcos Coronado, Jose Rodriguez

**Affiliations:** a Facultad de Ciencias de la Ingeniería y Tecnología, Universidad Autónoma de Baja California, Blvd. Universitario #1000, C.P. 21500 Tijuana, Mexico.; b Instituto de Ingeniería, Universidad Autónoma de Baja California, Blvd. Benito Juárez, Insurgentes Este, C.P. 21280 Mexicali, Mexico

**Keywords:** Virtual instrumentation, Diesel-biodiesel, Emissions, Virtual instrumentation, Compression ignition engine

## Abstract

This work shows the implementation and development of a set of virtual instruments focused on recording the physical parameters of a compression ignition engine that operates with diesel-biodiesel, 95% diesel and 5% soybean biodiesel. The components of the engine are constituted by several individual virtual instruments (VI) with the objective of registering parameters such as temperature (VITM), revolutions per minute (VIMRPM), fuel consumption (VIMFC), emission of gases such as oxygen (VIMO) and rust nitric (VIMNO). As a result of the research, the software development, hardware of each of the VIs is presented using the virtual programming platform Labview 2015®, the calibration of the O_2_ sensors, NO and the result of the operation of the engine at 850 rpm constant an ambient temperature of 25 ℃, a relative humidity of 40% and an operating temperature at the engine head of 65 ℃, obtain a fuel consumption of 0.0616 l/min and an average emission of O_2_ 10% and for the NO 540 ppm.

• Implementation of virtual instrument focused on evaluate the physical parameters of CI engine operate with diesel-biodiesel.
• The engine runs at 850 RPM under controlled conditions of 25 ℃ and a 40% RH with B5 mixture.
• Engine emissions are constant and stable at 10% Vol. O_2_ and 540 ppm of NO.

## Graphical abstract

**Figure:**
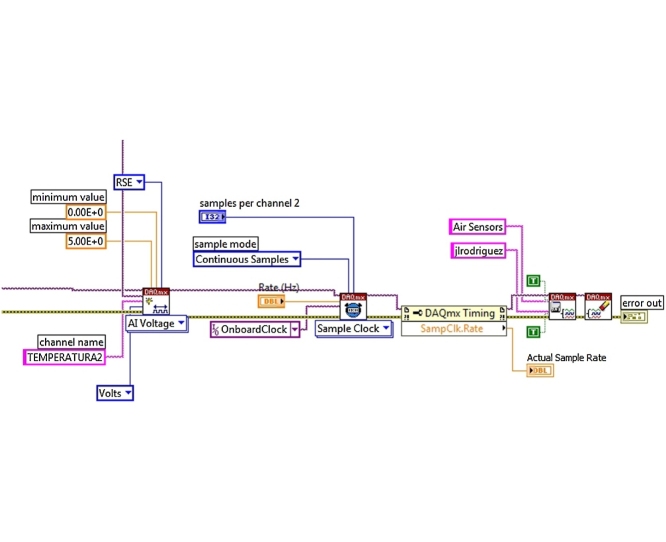

**Specifications Table**

**Table:** 

Subject Area:	*Engineering*
More specific subject area:	*Biodiesel*
Method name:	*Virtual instrumentation*
Name and reference of original method:	*If applicable, include full bibliographic details of the main reference(s) describing the original method from which the new method was derived.*
Resource availability:	*Lab View 2015, DAQ 6009 the National Instruments and Fluke 500A calibrator.*

## Introduction

The virtual instrumentation is a concept that include software and hardware systems that, with the use of a computer, substitute a measurement and monitoring instrument in the real world. Each program and hardware that fulfills this function is called Virtual Instrument (VI). Commercial systems, in most cases, the concept of VI is realized in an object-oriented programming language [1]. The modern scientific instrumentation boosts the introduction and development of systems based on the virtual instrumentation [[2], [3], [4], [5], [6], [7], [8]].

The advantages of the VI are the attached versatility that comes included in the software and hardware, the reduced cost by acquisition channel in comparison with traditional hardware instruments whose function is defined by the manufacturer, the ease of customization of the specific necessities of each user and the use of programming language [9].

The virtual instruments of monitoring and process control are used for their low cost, they combine a non-exclusive operation hardware with a powerful software and configuration flexibility; obtaining as a result a scalable architecture instrument, that is to say, with possibility to be adapted to the process whenever is required [[10], [11], [12]].

The virtual instrumentation involves some aspects like the measuring signal acquisition, as well as the processing, analysis, storage, distribution and deployment of data and information related with the physical phenomenon measurement, it also involves the human-machine interface, as well as the communication with other devices [7]. The present work has as objective the development and implementation of virtual instrumentation based on the Labview 2015® virtual programming platform for the characterization of the functioning of a diesel engine operated with diesel-biodiesel blends, and the development of virtual instruments.

### Existing systems to characterize the CIM operation

The development of the instrumentation and the test of the internal combustion engine (ICE) functioning have change considerably in the last decades, evolving from a purely mechanic functioning to a multidisciplinary functioning; incorporating knowledge branches like mechanic, electronic and computing, obtaining as objective the creation of engineer of hardware-software focused on the measurement, analysis and/or control of parameters that in influence in the proper function of a CIM, the instrumentation of any engine incorporates a broad range of sensors and actuators, with different types of signal and communication protocols [13,14].

The application of the virtual instrumentation for the measurement of gas emissions of internal combustion are currently realized through the use of autonomous modulation meters, dedicated and specialized, that provide information about the composition of the emitted gases by the engine combustion. For this reason, virtual systems that allow to measure and monitor the concentration of the emission gases in vehicles have been developed.

## Materials and method

### Virtual instruments used for the characterization of the ICE

**Fig. 1:**
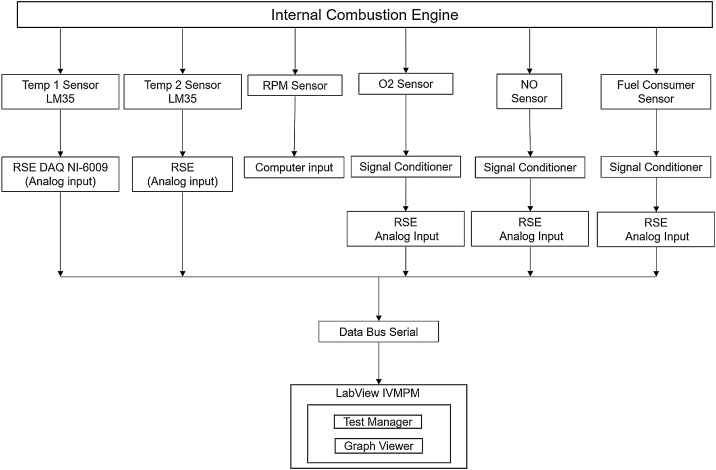
Operating block diagram of VI’s.

The hardware system used for the characterization of the ICE is constituted by the virtual instrument measurement of temperatures (VIMT), the virtual instrument for the measuring of revolutions per minute (VIMRPM), the virtual instrument for the measuring of fuel consumption (VIMFC), the virtual instrument for the measuring of oxygen (VIMO), and the virtual instrument for the measuring of nitric oxide (VIMNO). All the hardware and software developed for the characterization of the ICE is basically constituted by the virtual instruments (VI’s), signal conditioning, the data acquisition card (DAQ 6009), and the laptop (PC), as shown in Fig. 1.

For the acquisition and processing of data, the following devices were used: a data acquisition card, which is the one in charge to digitalize the information provided by the signal conditioners. A DAQ card was used; USB model 6009 with 8 analogue inputs, 14 bits, 48 kS/s, and 2 static analogue outputs of 12 bits; 12 digital inputs and outputs, and a 32 bits counter of National Instruments. The digital signals are transferred to the PC; model Lenovo Intel(R) Core (TM) i7 – 471CHQ CPU @ 2.50 GHz. Windows 10 operating system.

### General functioning of the analogous acquisition

**Fig. 2:**
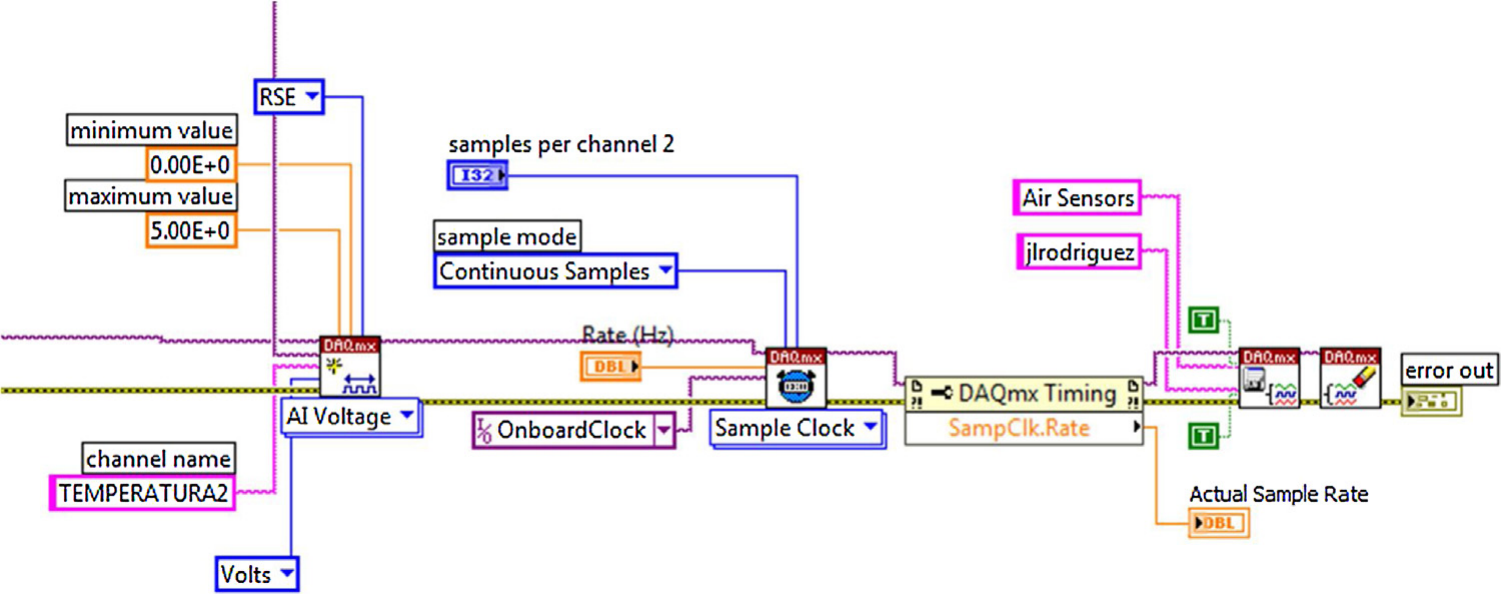
Parameters configuration for the tasks.

The analogous acquisition begins by defining the DAQ 6009 physical channels, defining the maximum and minimum voltages; to have a better use of the resources of the card, the “Reference signal ended” is used; the same configuration is used for all analogue inputs [15,16]. Once the channel is created the timing is set, selecting the sampling clock, the frequency, sampling mode, and the channel sample; once realized the aforementioned tasks, the procees to save the acquisition work with the name “Air sensor” in the IV called “Task build” as shown in the following Fig. 2.

**Fig. 3:**
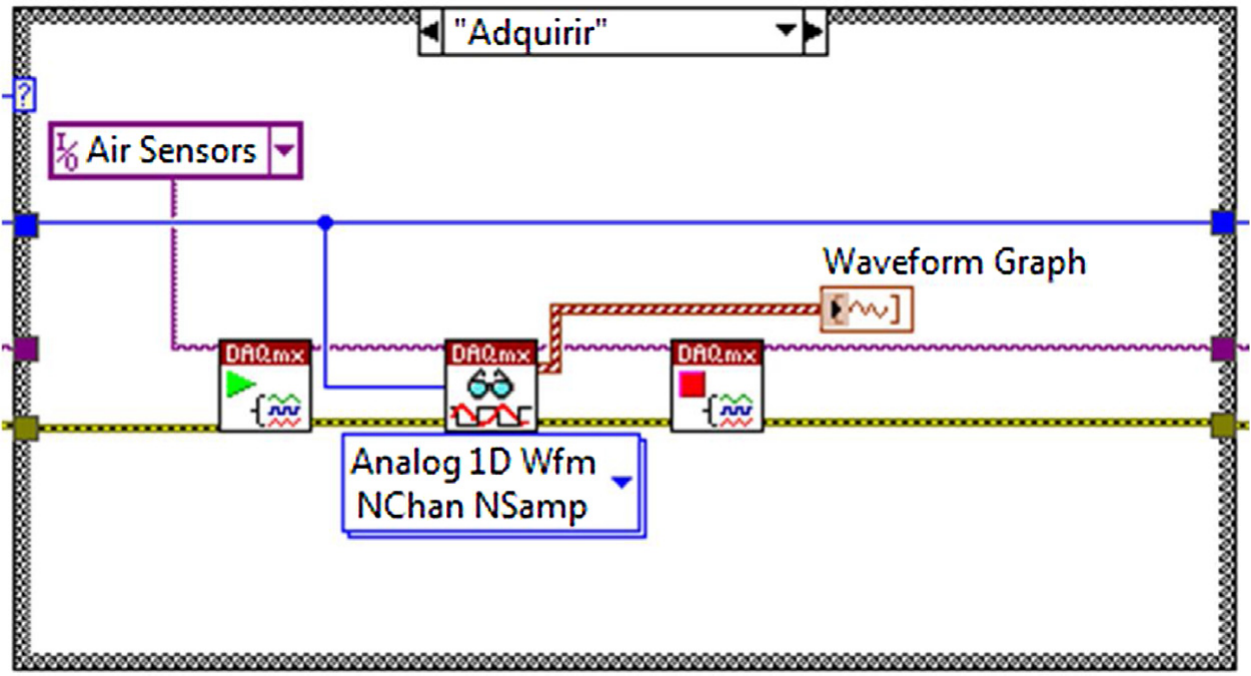
Analogue data reading.

Once the tasks are saved it is now possible to realize the “Case” of the acquisition of the data, initiating the measurement task by reading the analogue inputs and deploying the values In the “Waveform graph” indicator as shown in Fig. 3, subsequently the task is stopped to avoid an over flow in the “Buffers” when one wishes to end the application is necessary to abort the work.

### Virtual instrument for the measurement temperature (VIMT)

**Fig. 4:**
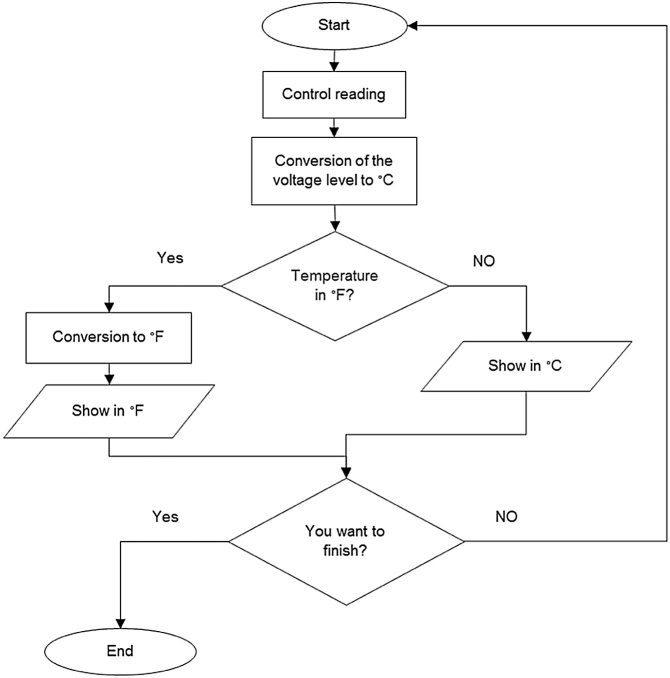
Operating block diagram of VIMT.

Since all sensors work with very low voltages and contemplating that the power of our acquisition data system works with a supply of 5 v. The VIMT is formed by a sensor in solid state at LM35 that offers a linear output signal with a scaling factor of 10 mV/°C, considering a range of temperatures lower than −55 °C with a conversion factor of −550 mV; and a range of temperatures higher than 150 °C with a conversion factor of 1500 mV, this range of temperature is enough to measure the temperatures presents in the whole systems. The Fig. 4 shows broadly the functioning of the VIMT in a block diagram.

**Fig. 5:**
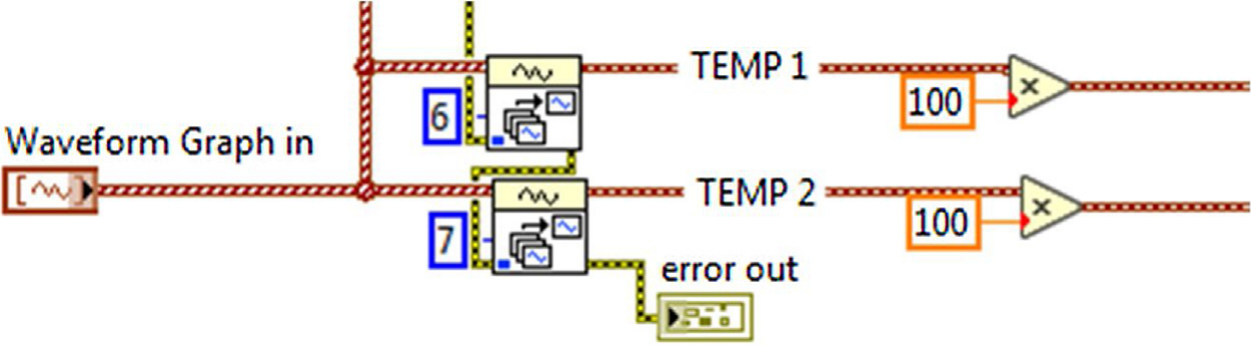
Voltage conversion to degrees.

**Fig. 6:**
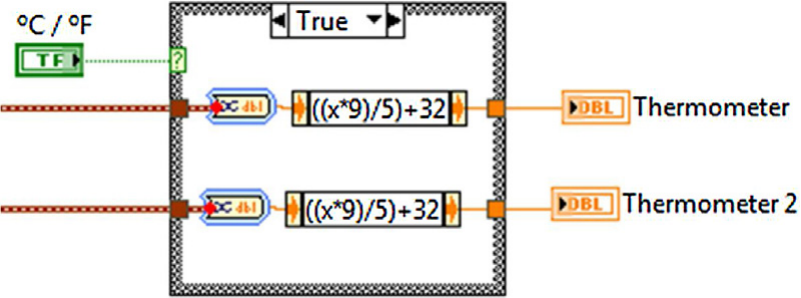
Centigrades conversion to farenheit.

For the physical connections between the temperature sensors, the amplifiers and the DAQ, a shielded cable was used for the sensors were exposed to vibrations, temperatures, electromagnetic waves and noise. To protect the circuit from the sensors connections with the DAQ card, a small circuit that uses a resistance of 10 KΩ was build. The program was designed for the use of two LM35 sensors, processing the information of the temperature recordings with the possibility of showing the temperature values in two different indicators, these measurements are shown in an indicator in the interface’s front panel. In Fig. 5 is shown the programming, where the different functions for the converting of the voltage levels to degrees centigrade are executed; likewise, in the code are incorporated functions for the conversion of °C to °F degree, showing the scale corresponding to the temperature see Fig. 6.

### Virtual instrument for the measurement revolutions per minute (VIMRPM)

**Fig. 7:**
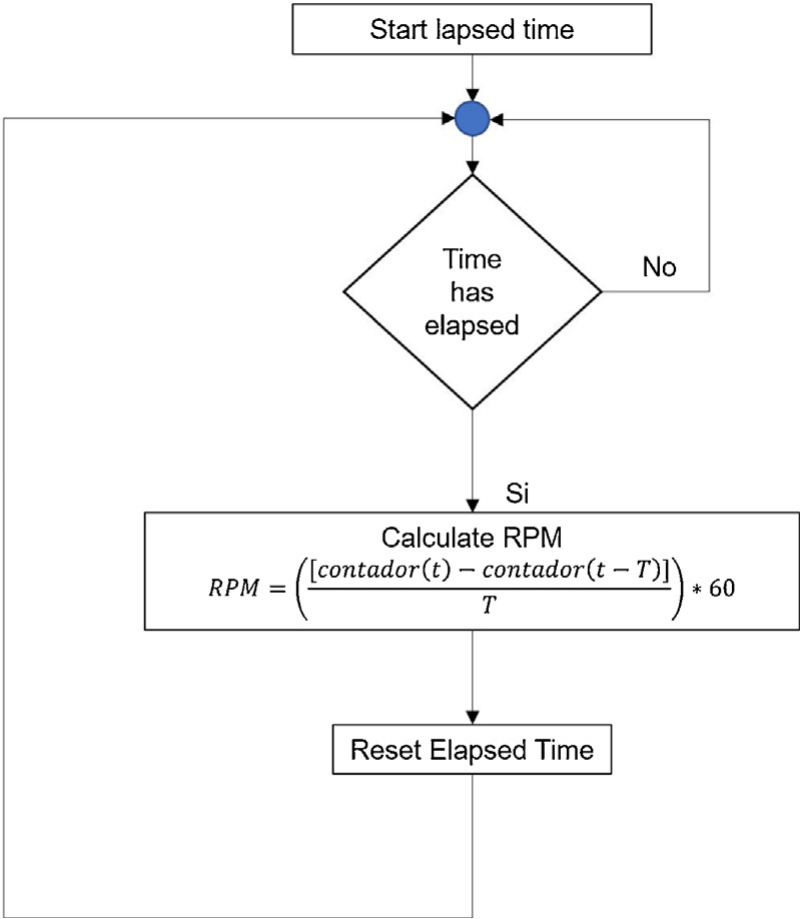
Operating block diagram of VIMRPM.

As part of the characterization of the diesel motor, it was developed a VIMRPM with the capacity to quantify the revolutions per minute the engine flywheel unit rotates, and thus registering the revolutions per minute the crankshaft rotates, for this using an infrared diode and a receiver diode which are one in front of the other at a non-maximum distance of 1.5 cm sending a continuous signal among them, sending a “low” or a “0 v”. The “Counter input” of the DAQ is used to read the pulse bursts generated by the receiver diode, detecting the rising edge of this signal, through this ascending counting indicated in the “Counter edge” the rpm calculation is realized, in Fig. 7 a block diagram that describes broadly the program functioning can be observed.

**Fig. 8:**
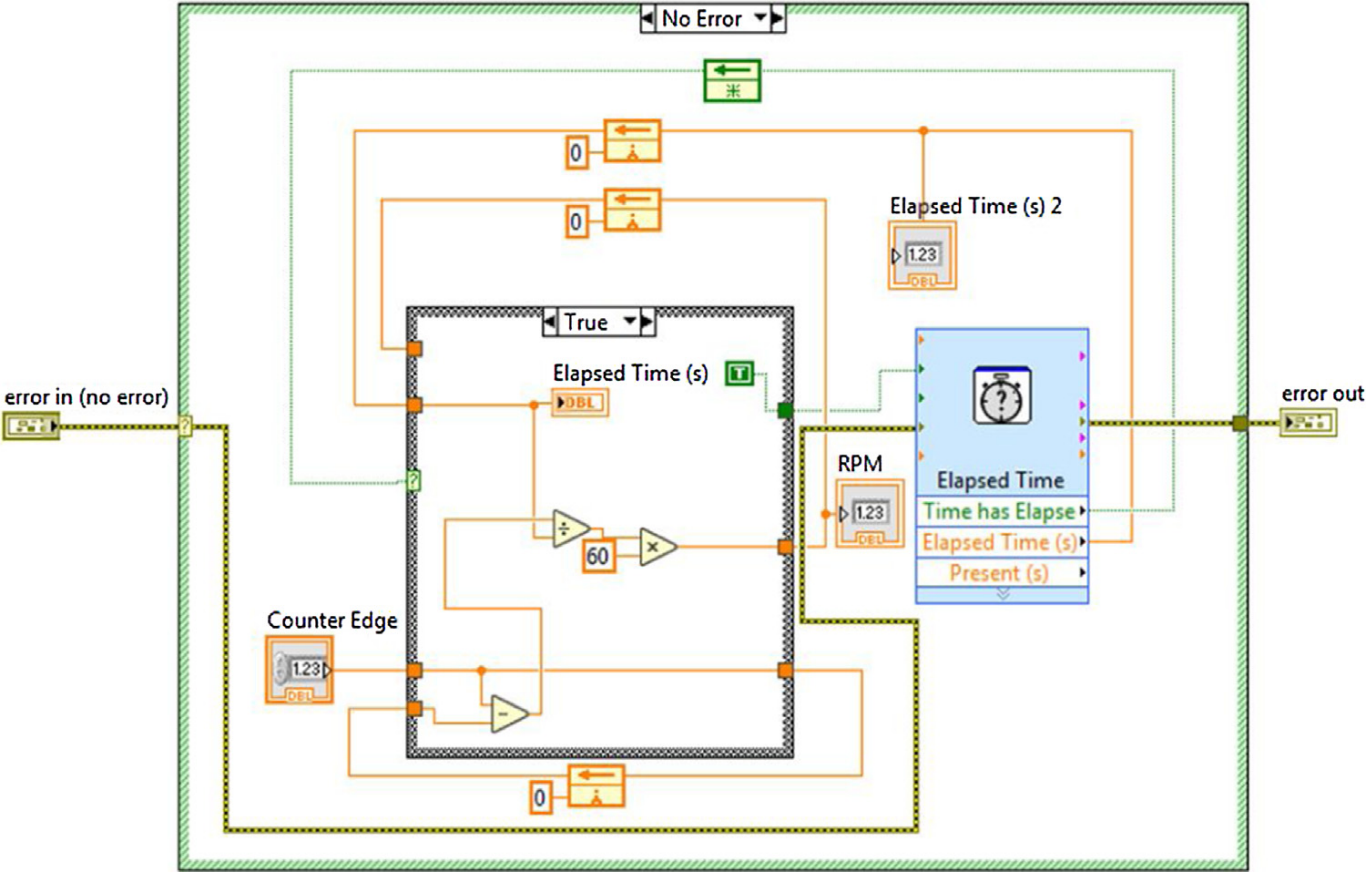
VIMRPM programing block.

In Fig. 8 is shown the implementation of the previous diagram using the programming platform LabView 2015 to calculate the VIMRPM of the engine crankshaft rotation [17,18].

### Virtual instrument for the measurement of fuel consumption (VIMFC)

**Fig. 9:**
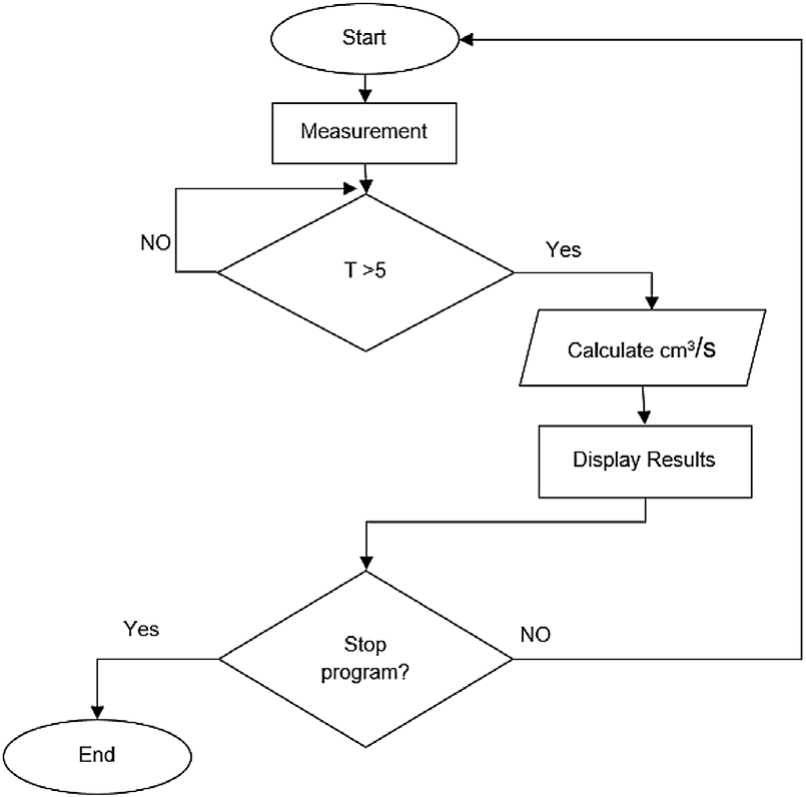
Operating block diagram of VIMFC.

For the development of the VIMFC was required to have the capacity to measure approximately 1.3 l, which is the estimated consume of the engine in an hour working at a constant speed of 850 RPM, that is the duration cycle of each test run. The system works basically with an ultrasonic sensor that sends a high frequency signal, which bounces in the object that is being measured and calculates the time the signal takes to come back to convert the time in distance. Based on these principles, a cube-like shaped container was built of polycarbonate with the purpose of having a specific design area of 12.2 × 11.1 cm to obtain a total area of 135.4 cm^2^, knowing the specific area of the container allowed to proceed to calibrate the system to add a correction factor to the programming, thus obtaining a final margin of error of +/−50 ml for each 1.5 l. The calculation for fuel consumption (FC) is indicated by Eq. (1), in Fig. 9 is shown a block diagram that describes broadly the functioning of the VIMFC program.

(1) FC =  [(12.2 cm* 11.1 cm) *(Au (t -5) cm –Au (t) cm))]/(5 s)

**Fig. 10:**
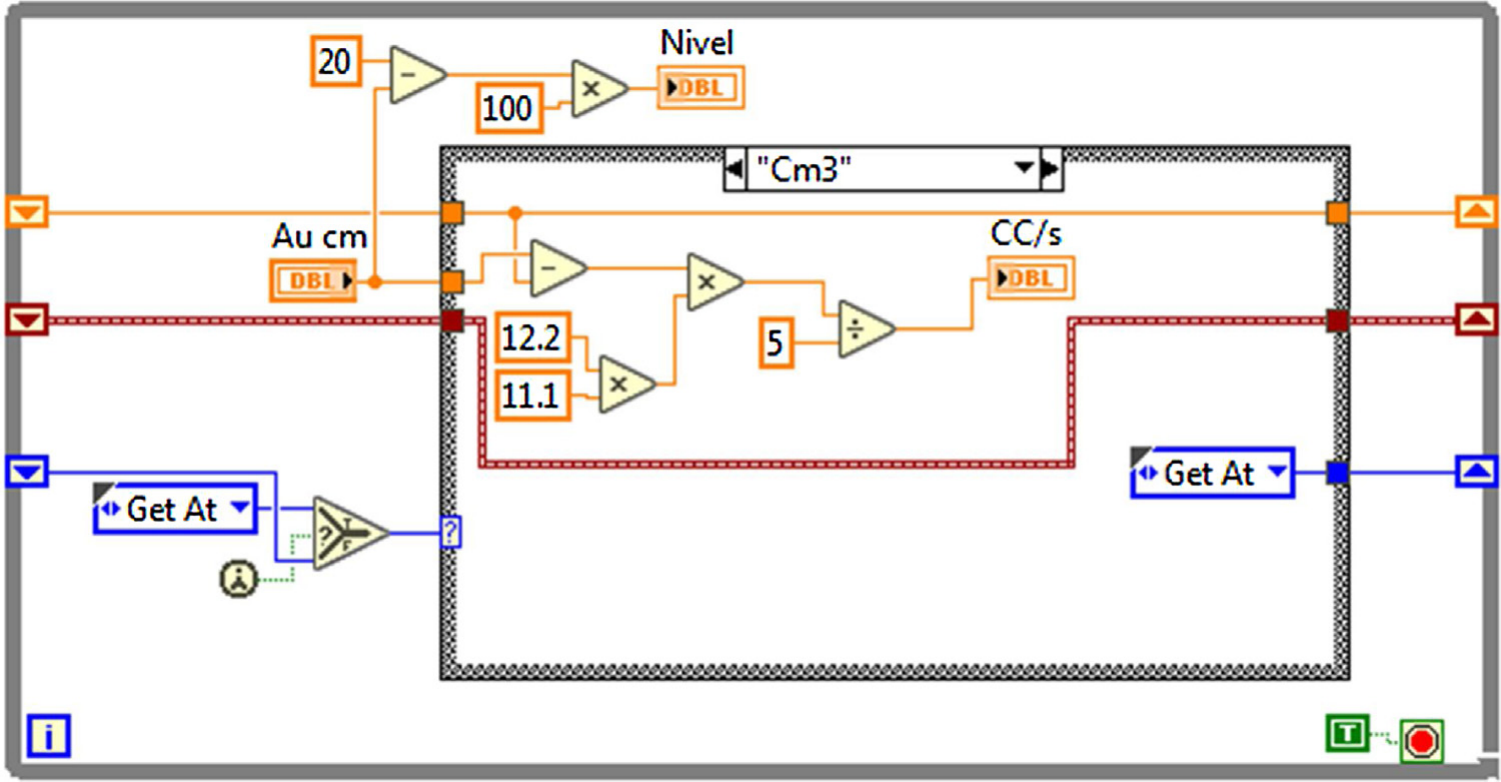
While loop VIMFC programing block.

The Fig. 10 presents the description of the programming of the main block’s that constitute the VIMFC. In the programming diagram can be observed an indicator that measures the fuel’s level inside the container; if the height of the fuel meets these conditions, the volume calculation is carried out in cm^3^/s unities, updating the indicator every 5 s, showing the results in two different indicators: level in ml, and consumption in cm^3^/s.

### Virtual instrument for measuring oxygen (VIMO)

For the development of the VIMO was required a sensor that had the capability to measure the oxygen percentage contained in the emission gases; such percentage ranges from 0 to a maximum of 30%. To carry out the VIMO’s development, the sensor model EC410 was used; Table 1 shows the technical parameters of the sensor [19]. It is an electrochemical sensor that detects the O_2_ gas concentration, quantifying the current produced by the sensor; the functioning of the sensor is based on the electrochemical principle, which consists in a working electrode found in the electrolytic cell; it works through an electrochemical oxidation process reacting with the gas to take census, the produced current in the census electrochemical reaction of the gas produces a direct proportion response with the O_2_ concentration, obtaining as a result the oxygen concentration percentage obtained through the current value. Since the current is too small to be processed by the DAQ, it was necessary to build a circuit incorporating a signal conditioning; recording an output voltage (Vo) of 2.435 V when 1% of oxygen concentration is registered, considering that it is the lowest concentration that can be registered in the sensor. The DAQ 6009 card process the O_2_ increases in percentage equivalent to 0.065 V/1%. The Eq. (2) was used to calculate the output voltage. Eq. (3) was implemented in the VI to convert the output voltage Vo with a range of 2.5–1.135 to the range of 0–21% of oxygen.

(2) *Vo* = 2.5 V − 65 mV/% * [O_2_%]

(3) O_2_% = −15.3846 Vo + 38.4615

**Table 1:** Technical parameters of the oxygen sensor.

Features	Operating conditions	
	NO	O_2_
Operating ranges	0–2500 ppm	0–30% O_2_
Model	EC4-2500-NO	EC410
Sensitivity	21–64 nA/ppm NO	N/A
Zero in air at 20 °C	−0.06 To 4.5 ppm NO	N/A
Zero deviation (−20 to +50 °C)	−2 to 10 ppm NO	N/A
Resolution	0.5 ppm NO	0.1% O_2_
Response time	t 90 = 35 s	t90 < 15s
Temperature range	−20 to +50 °C	−20 to +50 °C
Operating humidity	15–90% RH Non-condensing	15–95% RH
Pressure range	90–110 kPa	90–110 kPa
DC supply recommended	N/A	N/A

**Fig. 11:**
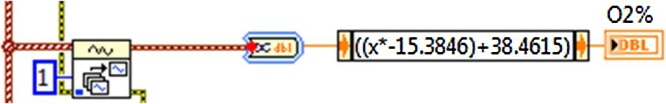
While loop VIMO programing block.

The virtual programming displayed in Fig. 11 shows how the measurement of the sensor’s voltage and their respective conversion factor to oxygen percentage in a range of 0–21% in O_2_ volume, with a resolution +/−0.1% takes place. The O_2_ percentage is shown in the program’s main screen indicators.

### Virtual instrument for the measurement of nitric oxide (VIMNO)

For the development of the VIMNO the sensor selecting presents the characteristics for the nitric oxide measuring in a percentage between the ranges of 0–2500 ppm of concentration, the sensor used is model EC4-2500-No, Table 1 shows the sensor’s technical parameters. As well as the oxygen sensor used for the measurement of nitric oxide is electrochemical, it was also required to make an integrated circuit contemplating a signal conditioner. Eq. (4) characterizes the measurement system and represents the output voltage (Vo) calculation. As output voltage Vo has a value of 2.508 V when 1 ppm of NO is registered; considering that it is the minimum concentration value registered. The data acquisition card registers increase with a value of 0.004 V/1 ppm of NO. The Eq. (5) was implemented in the VI to convert the output voltage Vo with a range of 2.5–10.5 v to the range of 0–1000 ppm of NO.

(4) *Vo* = 2.5 V + 8 mV/ppm*[*NO* ppm]

(5) *NO* ppm = 125 Vo – 312.5

**Fig. 12:**
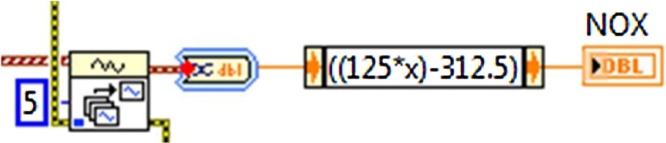
While loop VIMNO programing block.

The virtual programming displayed in Fig. 12 shows how the measurement of the voltage sensor and its respective conversion factor to ppm of nitric oxide with a range of 0–1000 ppm and a resolution of +/−0.5 ppm takes place. The ppm concentration is shown in the program’s main screen graphic display.

### Calibration of the O_2_ and NO sensors

With the purpose of corroborate and validate the proper function of the sensors a Fluk 500A calibrator, which is a multifunction calibrator traceable to the National Metrology Center in Mexico City, was used; with the objective of corroborating that the sensors indicates 0 V when is immersed in an atmosphere 100% of gas inert to the sensor, in this case was used oxygen with a purity level of 99% and nitrogen in gaseous state with purity of 99%. The test were realized under a controlled atmosphere in a temperature of 25 °C, relative humidity of 40% and atmospheric pressure of 1 atm, once immersed the sensor in the inert gas atmosphere, wait30 min with the finality that the sensor would stabilize. In Table 2 are shown the results of the sensors calibrations.

**Table 2:** Sensor’s currents values.

Sensor’s calibration			
Sensor	Calibration gas	% Gas	Answer (V)
O2	Nitrógeno	100	1.249
O2	Aire Ambiente	20.9	0.0014
NO	Oxigen	100	2.501

### Compression ignition engine characterization

The engine used for the VI’s development was a compression ignition engine (CIE), which uses diesel oil as fuel, 4-cylinder in line director injection and air supply at atmospheric pressure Mercedes Benz. The sensors used to measure the emissions produced by the diesel engine present error problems while taking census of gases with temperatures higher than 50 °C and percentage higher than 95% of relative humidity, to ensure a proper functioning of the sensors and, thus, for the acquisition of accurate information the temperature must not surpass 50 °C at the moment of contacting the sensors. There was a necessity to design and build a heat exchanger system (HES) used for the decrease of temperature of the gases from the engine’s exhaust manifold.

The HES contains the following elements:

1 Radiator with a heat transfer area of 50 × 100 × 5 cm
2 Water pump with a flow of 5.0 l/min.
3 Container with a storage capacity of 100 l.
4 Copper pipe of ¾” of diameter per 1/8” of thickness.
5 Two fans, one of 110 VCA at 1.5 A, and the second of 12 VCD at 3 A.

**Fig. 13:**
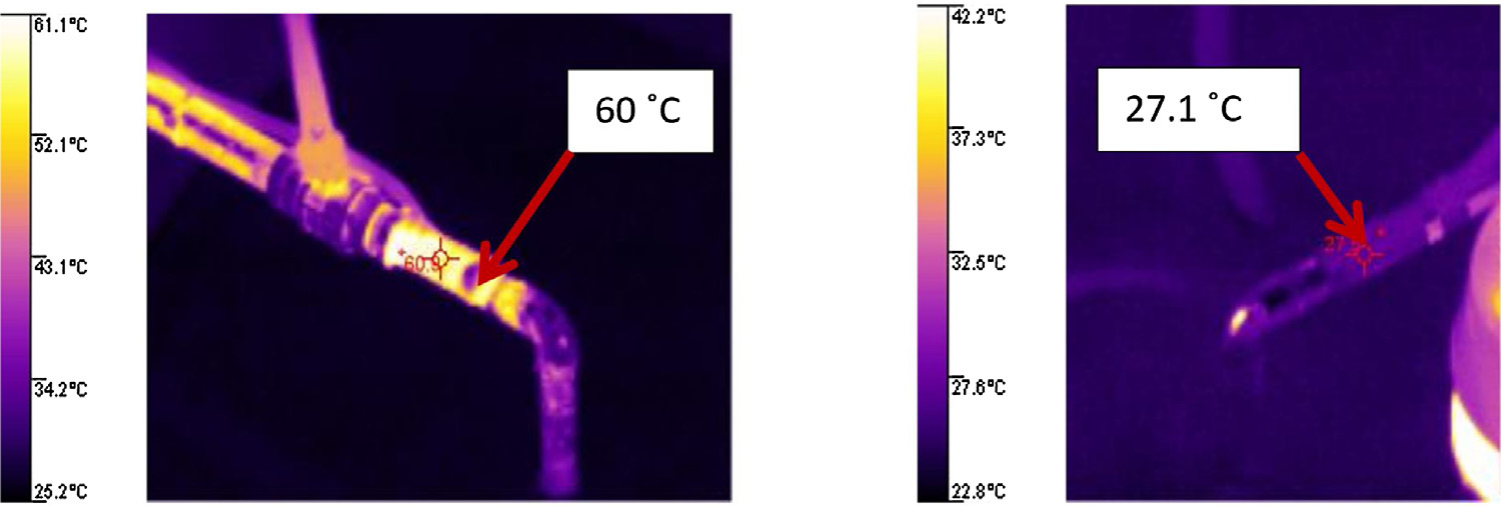
Heat exchanger inlet and outlet temperatures.

The CIE of the escape gases is constituted by a water bomb with a flow of 5 l/min used to maintain the water in the container with a capacity of 100 I in a closed circuit, making the water flow through the radiator which presents a transfer area of 50 cm × 100 cm × 5 cm, and using the two fans to generate an air forced fluid through the radiator, this air is in a controlled environment at 25 °C. The heater exchanger system reduced the temperature of the gases originating from the combustion realized by the CIM by an average of 60 °C registered at the coil inlet, and decreases its temperature to 35 °C approximately; this temperature is recorded at the copper coil’s outlet, as shown in Fig. 13, maintaining for 1.5 h the engine’s operation a temperature under the 50 °C, requirement necessary for the sensor’s proper functioning.

For the reduction of condensed water and steam there was a need to build a condensed water trap, three moisture traps and a single moisture trap placed before the gas enters the sensor; to ensure the retain of the all humidity quantity from the combustion gases, all moisture traps contain blue silica gel inside which works as a moisture saturation indicator when changing the silica color from blue, in the lack of humidity, to pink, when the silica is humid saturated.
