# Supplementary material for: Development and implementation of virtual instrumentation based on LabView applied to compression ignition engines operated with diesel-biodiesel blends
Source: MethodsX. 2019 Oct 4;6:2782–92. doi: 10.1016/j.mex.2019.09.024 (PMC6909051; doi:10.1016/j.mex.2019.09.024)
Supplement: Supplementary file 1 [file mmc1.docx]

**Supplementary material *and/or* Additional information:**

1. *Results*

*The systems were individually developed; experimental tests were realized using a diesel-biodiesel blend with 95% diesel and 5% biodiesel; the programs developed through Labview 2015® are presented below, however each VI fulfills a specific function: the VIMT’s main function is to measure on time the temperature in a specific engine area and the HES, denominated “Sensor 1”, quantifies the exhaust gases temperature before getting in contact with the O2 and NO sensors, the “Sensor 2” measures the heat exchanger’s exit gases temperature, “Sensor 3” measures the engine’s exhaust manifold gases, “Sensor 4” measures the engine’s head temperature; Figure 14 shows the main screen for the VIMT’s user. The program was designed for the use of for LM35 sensor, processing the temperature recording’s information and showing in the screen each second in °C values. In figure 15 are graphed the results of a functioning test for VIMT, operating the CIM with a blend fuel [17].*

*
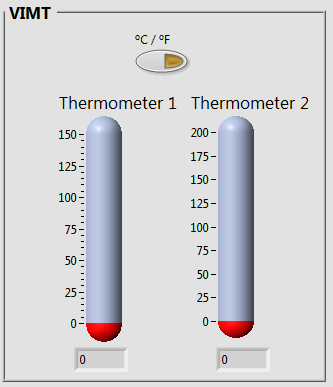
*

*Figure 14: Graphic user interface for VITM.*

*Figure 15: Graphical interface for the VIMT user.*

*In figure 16 is presented the graphic user interface that the VIMRPM presents, the main function is to measure the crankshaft rotations in a time frame, specifically per minute. Figure 17 presents the graphic users’ interface of the VIMFC, where is displayed the height of around “0 cm” registered by the ultrasonic sensor, an alarm lights indicating “Full tank”; when the ultrasonic sensors indicates a value of “25 cm”, an alarm lights instantly that indicates “Out of fuel”, the VIMFC shows the fuel consumption in percentage, liters and cm3.*

*
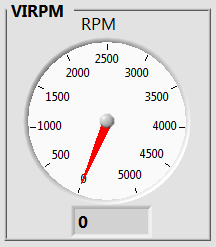
*

*Figure 16: Graphic user interface for the VIMRPM.*

*
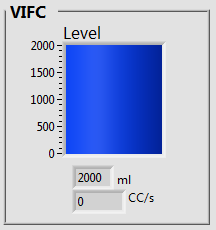
*

*Figure 17: Graphic user interface for the VIMFC.*

*The graph presented in figure 18 is the result of a VIRPM and VIMFC performance test plotted over time, this test was performed when the CIM was operating with B5.*

*Figure 18: Graphic user interface of VIRPM and VIMFC.*

*Figure 19 shows the graphic interface for the VIMO’s user, which displays the oxygen concentration percentage contained in the exhaust gases; the user has the option of stopping the data acquisition. In figure 20 is displayed the VIMO’s obtained results graphic [21, 22].*

*
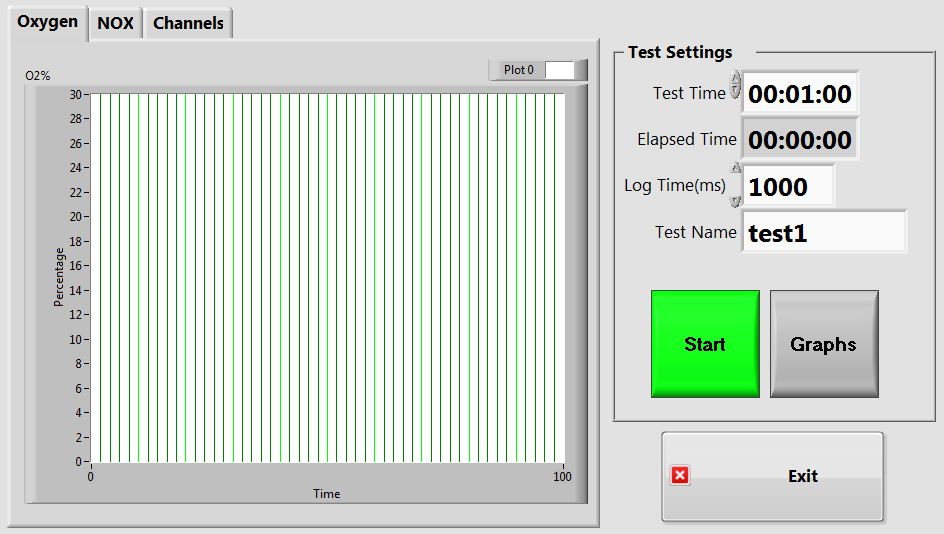
*

*Figure 19: Graphic user interface for O2 concentration measurement.*

*Figure 20: Graph of O2 concentration measurement results.*

*In figure 19 is displayed the VIMNO user graphic interface, which shows the percentage of carbon dioxide in a range of 0 to 2500 ppm; this information is translated into hexadecimal format and delivered through the serial port directly connected to the PC to include it in the VIMNO, finally showing the VIMNO front panel and the controls to configure the serial communication, the percentage of concentration vs. time data are graphed in real time, as well as the emissions results. Figure 21 shows the graphic of the VIMNO tests obtained results.*

*Figure 21: Graphic user interface for IVMNO.*

1. *Conclusions*

*The software and hardware used in the development of VITM, VIMFC, VIMREPM, VIMO and VIMNO, with the characteristic of compression ignition engines that use diesel-biodiesel blends, integrate a flexible system and a scalable tool to evaluate the performance of internal combustion engines that use conventional fuels, biofuels or mixtures. The results have been processed with acceptable precision, reliability and speed, showing a good performance and that the results are within the parameters set for the calibration equipment Fluke 500A calibrator, in general the development of the VI represents an acceptable cost compared with the cost of dedicated equipment for the census of each one of the variables of the CIE, this being an advantage for the users. If a larger capacity DAQ is used, the speed of data processing can be increased and the number of channels increased. output, translating in the increase of the variables to be measured. The system presented is not limited to engines of a specific capacity, on the contrary, it has the flexibility to adapt to different CIEs, for which the following aspects must be considered: operating capacity, operating conditions and the fuel used. The suppliers of this product are compatible with the temperature from 0 to 200 ℃, from 0 to 6000 for the rpm, since the CO emissions can be evaluated up to 2500 ppm and 100% of O2. A result of the tests of an operating temperature at the engine head from 65 ℃ to 850 rpm, an ambient temperature of 25 ℃ and a relative humidity of 40%, the fuel consumption of the engine during 30 minutes of operation of 1850 ml, an average emission generated with 10% oxygen and 540 ppm for NO. The system allows to visualize the saved data of the tests carried out, allowing the analysis, comparison and import of the results obtained from the test.*
